# Lisinopril increases lung ACE2 levels and SARS-CoV-2 viral load and decreases inflammation but not disease severity in experimental COVID-19

**DOI:** 10.3389/fphar.2024.1414406

**Published:** 2024-07-12

**Authors:** Yasmin Silva-Santos, Roberta Liberato Pagni, Thais Helena Martins Gamon, Marcela Santiago Pacheco de Azevedo, Mônica Bielavsky, Maria Laura Goussain Darido, Danielle Bruna Leal de Oliveira, Edmarcia Elisa de Souza, Carsten Wrenger, Edson Luiz Durigon, Maria Cecília Rui Luvizotto, Hans Christian Ackerman, Claudio Romero Farias Marinho, Sabrina Epiphanio, Leonardo José Moura Carvalho

**Affiliations:** ^1^ Laboratory of Malaria Cellular and Molecular Immunopathology, Faculty of Pharmaceutical Sciences, Department of Clinical and Toxicological Analysis, University of São Paulo, São Paulo, Brazil; ^2^ Laboratory of Malaria Research, Oswaldo Cruz Institute, Oswaldo Cruz Foundation, Rio de Janeiro, Brazil; ^3^ Immunology Laboratory, Heart Institute, Faculty of Medicine, University of São Paulo, São Paulo, Brazil; ^4^ Laboratory of Clinical and Molecular Virology, Institute of Biomedical Sciences, Department of Microbiology, University of São Paulo, São Paulo, Brazil; ^5^ Laboratory of Experimental Immunoparasitology, Institute of Biomedical Sciences, Department of Parasitology, University of São Paulo, São Paulo, Brazil; ^6^ Hospital Israelita Albert Einstein, São Paulo, Brazil; ^7^ Unit for Drug Discovery, Department of Parasitology, Institute of Biomedical Sciences, University of São Paulo, São Paulo, Brazil; ^8^ School of Veterinary Medicine of Araçatuba, São Paulo State University, São Paulo, Brazil; ^9^ Physiology Unit, Laboratory of Malaria and Vector Research, National Institute of Allergy and Infectious Diseases, Rockville, MD, United States

**Keywords:** SARS-CoV-2, COVID-19, K18-hACE2, angiotensin-converting enzyme inhibitors, ACEi, lisinopril

## Abstract

COVID-19 causes more severe and frequently fatal disease in patients with pre-existing comorbidities such as hypertension and heart disease. SARS-CoV-2 virus enters host cells through the angiotensin-converting enzyme 2 (ACE2), which is fundamental in maintaining arterial pressure through the renin-angiotensin system (RAS). Hypertensive patients commonly use medications such as angiotensin-converting enzyme inhibitors (ACEi), which can modulate the expression of ACE2 and, therefore, potentially impact the susceptibility and severity of SARS-CoV-2 infection. Here we assessed whether treatment of ACE2-humanized (K18-hACE2) mice with the ACEi Lisinopril affects lung ACE2 levels and the outcome of experimental COVID-19. K18-hACE2 mice were treated for 21 days with Lisinopril 10 mg/kg and were then infected with 10^5^ PFU of SARS-CoV-2 (Wuhan strain). Body weight, clinical score, respiratory function, survival, lung ACE2 levels, viral load, lung histology, and cytokine (IL-6, IL-33, and TNF-α) levels were assessed. Mice treated with Lisinopril for 21 days showed increased levels of ACE2 in the lungs. Infection with SARS-CoV-2 led to massive decrease in lung ACE2 levels at 3 days post-infection (dpi) in treated and untreated animals, but Lisinopril-treated mice showed a fast recovery (5dpi) of ACE2 levels. Higher ACE2 levels in Lisinopril-treated mice led to remarkably higher lung viral loads at 3 and 6/7dpi. Lisinopril-treated mice showed decreased levels of the pro-inflammatory cytokines IL-6 and TNF-α in the serum and lungs at 6/7dpi. Marginal improvements in body weight, clinical score and survival were observed in Lisinopril-treated mice. No differences between treated and untreated infected mice were observed in respiratory function and lung histology. Lisinopril treatment showed both deleterious (higher viral loads) and beneficial (anti-inflammatory and probably anti-constrictory and anti-coagulant) effects in experimental COVID-19. These effects seem to compensate each other, resulting in marginal beneficial effects in terms of outcome for Lisinopril-treated animals.

## 1 Introduction

COVID-19, discovered in December 2019 in Hubei province (China), is responsible for more than 6 million deaths worldwide (Zhu et al., 2020; Medicine, 2023). It is caused by SARS-CoV-2, a coronavirus (CoV) like SARS-CoV responsible for outbreaks of acute respiratory syndrome in Guangdong (China) in 2002–2004 (Ksiazek et al., 2003; Cui et al., 2019) and MERS-CoV discovered in the Middle East in 2012 (Zaki et al., 2012; De Wit et al., 2016; Su et al., 2016).

SARS-CoV-2 has a simple RNA structure surrounded by an envelope, nucleocapsid, membrane and spike protein. The latter plays a fundamental role in viral entry into the host cell (Bhalla et al., 2021), and is responsible for the viral binding to the angiotensin-converting enzyme 2 (ACE2). The spike protein is cleaved and activated by transmembrane serine protease 2 (TMPRSS2), completing adsorption, membrane fusion and genetic material release. On the other hand, ACE2 plays an essential role in regulating blood pressure through the renin-angiotensin system (RAS) (Hoffmann et al., 2020; Wang et al., 2020). RAS has several factors that make up a cascade of events to regulate blood pressure through the balance of products obtained by the degradation of angiotensinogen by renin, an enzyme produced in the kidneys, giving rise to the decapeptide angiotensin I (AngI). In the classical RAS pathway, AngI is cleaved by the angiotensin-converting enzyme (ACE) generating the octapeptide angiotensinogen II (AngII) as its product, AngII binds to the AT1R receptor, increasing blood pressure, producing a vasoconstrictive, apoptotic, pro-thrombotic effect and stimulating pro-inflammatory factors (Choi et al., 2020; Vaduganathan et al., 2020). In addition to the classical pathway, RAS presents an alternative pathway, in which the nonapeptide Angiotensin 1–9 (Ang 1–9) is produced by the cleavage of AngI by ACE2 and, again, cleaved into Angiotensin 1–7 (Ang 1–7) by ACE. Ang1-7 binds to the MAS receptor, reducing blood pressure and inflammation and producing a vasodilatory effect (Bhalla et al., 2021). Since ACE2 plays a key role in the homeostasis of the vascular system, regulating blood pressure and other functions, its binding to SARS-CoV-2 may have deleterious effects on the vasculature.

The balance in the RAS can be decisive in the development of severe forms of COVID-19, especially for risk groups such as hypertensive patients and those with cardiovascular diseases (Fang et al., 2020; Sommerstein et al., 2020). ACE2 is a molecule expressed in various body tissues, such as the epithelium of the lungs, including in the alveoli where it is abundant, in endothelial cells, in the heart, intestine, kidneys, brain and other organs (Uhlén et al., 2015).

The high expression of ACE2 in the upper airways and lung alveoli causes the virus to proliferate preferentially in these tissues, leading to an intense inflammatory response, diffuse alveolar destruction, loss of respiratory capacity, fibrosis and, frequently, the patient’s death (Li et al., 2015a; Wu, 2020). The damage caused by SARS-CoV-2 infection, resulting from apoptotic/necrotic events, induces hypoxia, vascular congestion and obstruction of the vasculature resulting from thrombotic events characterized by fibrin deposition. The mechanism responsible for the outcome of the disease may be related to the binding of the ACE2 receptor by SARS-CoV-2 and the invasion of target cells.

SARS-CoV-2 promotes a substantial decrease in the expression of ACE2 itself, fundamental for vascular function, increasing the levels of AngII (vasoconstrictor, pro-inflammatory, pro-thrombotic) and decreasing the levels of Ang1-7 (vasodilator, anti-inflammatory, anti-thrombotic). Decreased ACE2 expression can lead the body towards a constrictive and thrombotic bias, reducing blood perfusion and leading to an ischemic process, contributing to tissue damage in several organs (DIAZ, 2020; Li et al., 2003; Wu, 2020).

This situation causes ACE2 to have a dual behavior in COVID-19: it is the gateway for SARS-CoV-2 into cells, causing infection and, on the other hand, it causes a decrease in its expression and profoundly impacts the regulation of vascular function, generating systemic damage, which can worsen the disease (Diaz, 2020; Tignanelli et al., 2020; Liu et al., 2021).

Medications capable of modulating ACE2 expression, such as angiotensin-converting enzyme inhibitors (ACEi) or angiotensin receptor blockers (ARB) could produce deleterious effects by increasing ACE2 expression and therefore increasing viral load in the lungs. On the other hand, they could help preventing or decreasing the vascular consequences of ACE2 depletion by positively regulating ACE2 expression (Li et al., 2015a; Snyder and Johnson, 2020).

Initially, the use of ACEi or ARB was associated with worsening clinical outcomes in patients, as well as interruption of treatment after confirmation of the diagnosis of COVID-19 (Fang et al., 2020; Guo et al., 2020; Sommerstein et al., 2020; Soria Arcos et al., 2020; Vallejo Ardila et al., 2020; Vieira, 2021). Furthermore, it has been described that the treatment exposes patients to a high risk of developing moderate to severe forms of COVID-19 (Najafi et al., 2023; Walia, 2023).

On the other hand, Lopes and colleagues tested the impact of discontinuation *versus* maintenance of chronic ACEi/ARB therapy in patients with COVID-19, and no differences were found between the continued and discontinued therapy groups (Lopes et al., 2021). The risk of mortality was lower in hypertensive patients hospitalized with COVID-19 who used ACEi or ARB (Zhang et al., 2020; Dambha-Miller et al., 2023) and clinical manifestations were reduced in patients who used ACEi and no increase in severity was observed in patients hospitalized in intensive care unit (Hippisley-Cox et al., 2020; Armstrong et al., 2021).

Few studies in experimental models have been conducted to understand the mechanisms that may affect the RAS during COVID-19 infection. Evidence indicates that monotherapy with ACEi and/or ARB can increase ACE2 levels in the lung, small intestine, kidney and brain (Ferrario et al., 2005; Li et al., 2015b; Brooks et al., 2022). Therefore, this study aimed to determine whether the ACEi Lisinopril alters the quantification of ACE2 and whether they affect lung damage and the outcome of experimental COVID-19. We used the murine experimental model for COVID-19 treated with Lisinopril to address this question. This medication is used in patients with hypertension, congestive heart failure, acute myocardial infarction, kidney complications and *Diabetes mellitus* (Warner and Rush, 1988; Alderman, 1996; Pylypchuk, 1998; Brilla et al., 2000; Bezalel et al., 2015).

## 2 Methods

### 2.1 Transgenic mouse model for studying SARS-CoV-2 infection

The B6.Cg-Tg (K18-ACE-2)2Prlmn/J-strain 034860 animal model was used, which has a C57BL/6 background and expresses human ACE2 (hACE2) through a cytokeratin 18 (K18) promoter, mainly in respiratory airway epithelial cells (McCray et al., 2007; Yang et al., 2007; Moreau et al., 2020; Yinda et al., 2021). Animals were kindly provided by the Institute of Science and Technology in Health of the Fundação Oswaldo Cruz (ICTB-Fiocruz), Rio de Janeiro, Brazil.

### 2.2 Ethics

Ethics Committees for the Use of Laboratory Animals of the Faculty of Pharmaceutical Sciences/University of São Paulo (FCF/USP) and the Institute of Biomedical Sciences/USP (ICB/USP) approved this study with the license numbers 622 and 3858020621, respectively. All protocols followed the ethical principles of animal experimentation, recommended by Federal Law 11.794/2008 and the National Council for Animal Experimentation (CONCEA). The Internal Biosafety Committee (CIBIO-0092021/FCF) approved this study. Activities involving viral manipulation, animal infection and sample collection were carried out in a Biosafety Level 3 Laboratory (BSL3).

### 2.3 Treatment

Lisinopril (Medley 10 mg–Brazil registration 101810399008-7) was administered for 21 consecutive days to simulate continuous treatment (Brooks et al., 2022) and up to 7 days post infection. The mice were divided into four groups: Untreated Uninfected (UU), Lisinopril-treated Uninfected (LU), Untreated Infected (UI) and Lisinopril-treated infected (LI). Lisinopril was added to flavoured gelatin, which made the product palatable for voluntary ingestion. Daily, animals in the Lisinopril-treated infected and Lisinopril-treated Uninfected groups were treated with 10 mg/kg/day of Lisinopril, while animals in the Untreated Uninfected and Untreated Infected groups received gelatin without medication. The animals used in this study received treatment when they were 8/9 weeks old and were infected with SARS-CoV-2 when they were 11/12 weeks old. A preliminary experiment with 18 mice was performed to determine how the Lisinopril treatment affected lung ACE2 levels in healthy, uninfected animals. In the survival experiments, 38 mice were used. And a total of 30 mice per group (Untreated Uninfected, Untreated Infected and Lisinopril-treated infected) were used in the experiments to assess clinical scores, lung function, ACE2 levels, viral load, cytokine levels and histopathology. Not all animals were used in all measurements in all three timepoints, so specific information on sample size is provided in the figure legends. In all experiments, an equal number of male and female animals was used per group.

### 2.4 Virus and infection

After 21 days of treatment, mice were infected according to a previously described protocol (da Silva Santos et al., 2023). Professor Edison Durigon’s team from the Department of Microbiology at ICB/USP expanded and provided the virus according to the protocol established by Araújo and collaborators (Araujo et al., 2020). Mice were lightly sedated with isoflurane and infected intranasally with 10^5^ PFU of SARS-CoV-2 (original Wuhan strain: SARS.CoV2/SP02.2020.HIAE.Br).

### 2.5 Euthanasia and sample collection

Animals were euthanized with an anesthetic combination of Ketamine (150 mg/kg) and Xylazine (15 mg/kg) intraperitoneally, followed by exsanguination by cardiac puncture and blood collection. Subsequently, cardiac perfusion was performed (PBS 1x) following the protocol previously described (Gage et al., 2012). Three time points (3, 5, and 6/7 days post-infection) were defined to monitor viral load, lung capacity and ACE2 quantification.

Samples collected for viral quantification by qRT-PCR were subsequently maintained in RNA later (Invitrogen/cat.7021). For ELISA analysis, samples were maintained in radioimmunoprecipitation assay buffer (Thermo Fisher/89900) and protease inhibitors (Sigma/cat.S8830-20TAB). All these samples were quickly stored and kept at −80°C. For histopathology, fragments of the same tissues were collected in 10% formaldehyde and kept in 70% alcohol until processing.

### 2.6 Protein extraction

The tissues were macerated and subsequently inserted into Radioimmunoprecipitation assay buffer (Thermo Fisher/cat. 89900) and protease inhibitor cocktail (Sigma-Aldrich Cat. 58830) and homogenized using a pipette and tip. The samples were centrifuged for 5 min at 2,000 revolutions per minute (rpm) at 4°C, and the supernatants were transferred to other 1.5 mL microtubes, followed by new centrifugation for 20 min at 12,000 rpm. The supernatants were transferred again to new microtubes and stored at −80°C until processing. Pierce BCA protein assay kit (Thermo Fisher—Cat. 23225) was used to quantify the total protein of each sample, following the manufacturer’s instructions, and quantified on a Multiskan™ FC Microplate Photometer (Thermo Fisher—Cat. 51119000).

### 2.7 Quantification of ACE2 in tissue samples

Detection and quantification of ACE2 protein in tissues by ELISA (Enzyme-Linked Immunosorbent Assay) were performed using the Human ACE2 DuoSet ELISA kit (R&D Systems- Cat. DY933-05), following the manufacturer’s recommendations. ELISA plates were coated with 100 µL anti-ACE2 antibody (2 μg/mL diluted in PBS pH 7.4) overnight at room temperature (RT). The plates were washed 3 times with 300 µL of washing buffer (PBS + 0.05% Tween20) and blocked with 300 µL of blocking buffer (1% BSA in PBS, pH 7.4) for 1 h at RT. After blocking, plates were washed three times, and 100 µL of standards or samples containing 100 μg/mL of total protein were inserted into each well. In some cases, samples were diluted in dilution reagent (and assigned their respective correction factors in the result analysis) provided by the kit and incubated for 1 h at RT. Then, the plates were washed, and 100 µL of the detection antibody (100 ng/mL diluted in 1% BSA in PBS, pH 7.4) was added for another hour. After a new washing step, 100 µL of secondary antibody (HRP) conjugate were added for 1 hour at RT. Subsequently, the plates were washed and 100 µL of substrate (TMB) were added to the wells. Colorimetric reaction was stopped by adding 50 µL of 1 M sulfuric acid and subsequently analyzed using the filter at 450 nm.

### 2.8 RNA extraction

For SARS-CoV-2 viral load quantification, the inferior lobe of the right lung (weighting −50 mg) was used. Samples were macerated with the MagNA Lyser equipment (Roche Diagnostics, Mannheim, Germany) and centrifuged at maximum speed for 15 min. A 150 μL aliquot of the supernatant was mixed with 200 μL of Lysis Buffer (BioMerieux, Lyon, France). Then, total nucleic acid extraction was performed according to the protocol established by the equipment manufacturer Nuclisens MagMax (BioMerieux, Lyon, France).

### 2.9 Virus detection

The genetic material extracted was subjected to RT-qPCR diagnostics to detect SARS-CoV-2 and mammalian Ribonucleoprotein (RNP), used as an extraction control. For this, the AgPath-ID™ One-Step RT-PCR kit (Applied Biosystems/Life Technologies, Austin, Texas, United States) and the 7500 Real-Time Systems equipment (Applied Biosystems/Life Technologies, California, United States) were used, with protocols adapted from Corman et al. (2020). Both forward, reverse, and probe primers detecting the SARS-CoV-2 spike and RNP were used in equal proportions and concentration of 10 µM. As negative and positive controls, Nuclease Free Water and a clinical isolate in Vero-E6 cell culture, duly tested and standardized, were used in all reactions (Corman et al., 2020). Finally, the cycles were: 45°C for 15 min, once; 95°C for 10 min, once; 95°C for 15 s and 57°C for 1 min, 45 times. In diagnosing, samples with Ct values < 37.99 were classified as positive, and the number of copies per mL determined in relation to the standard curve of the positive control, whereas those with Ct values > 42 were categorized as negative. Samples with values falling between 38 and 41.99 were deemed inconclusive and underwent repeated testing starting from the total nucleic acid extraction stage. After virus detection via RT-qPCR, the viral load in the samples was quantified in copies/mL. The results showed a range between 4.65 × 102 and 4.41 × 109 copies/mL (da Silva Santos et al., 2023).

### 2.10 Assessment of lung capacity

To evaluate the respiratory pattern, mice were placed in plethysmographic chambers (BUXCO Electronics, United States) on days 3, 5, and 6/7 after infection. Whole-body plethysmography (WBP) evaluated the respiratory capacity of non-anesthetized animals by recording pressure changes reflected in waves proportional to respiratory flow for 10 min without movement restrictions. The equipment measured several parameters, including respiratory frequency (RF), tidal volume, enhanced pause (Penh), and expiratory flow curve (Rpef).

RF is the number of complete breaths per minute, Penh is the wave product of the expired respiratory peak (PEP) in relation to the inspiratory peak (PIP), obtained from the respiratory pause calculated from the expiratory time (Te) under the expiration time of 65% of the air volume, minus 1 (Pause = Te/Rt−1). This result is multiplied by the PEP below the inspiratory peak PIP (Penh = pause×PEP/PIP). The tidal volume assesses adequate lung ventilation and is measured by the proportion of inspired and exhaled air (in mL) in each respiratory cycle. Rpef is the ratio between PEP/Te. Like Penh, Rpef is an indicator of constriction (Lomask, 2006; Malhotra, 2007; Menachery et al., 2015; Hallett S et al., 2021).

### 2.11 Clinical evaluation

Mice were monitored daily, behavioral changes were observed (lethargy, difficulty breathing, hunched posture, piloerection, tremors, exudate around the eyes/nose, eye closure, and death) and recorded in an individual score table, according to the intensity of symptoms (scores, with values from 0 to 4, with 0 being absence of clinical signs, 1 reported for mild signs and 4 for severe signs). Table and score were prepared following the recommendations of the Guide for implementing the Humanitarian End point of the Federal University of São Paulo (UNIFESP, 2020). Signs of pain were also monitored according to the Grimace Scale for mice (Langford et al., 2010). A humane endpoint was established with criteria such as weight loss (>20%), animal inactivity and no response to external stimuli.

### 2.12 Cytokine quantification in serum

IL-6 (Thermo Fisher—cat.88-7064-88), TNF-α (Thermo Fisher—cat.88-7324-88) and IL-33 (Thermo Fisher—cat.88-7333-88) were quantified in serum and lung at the last time point, according to the manufacturer’s instructions (Thermo Fisher—cat.88-7064-88). ELISA plates were coated with each cytokine (100 µL) overnight. Plates were washed 3 times with 300 µL of washing buffer and block (1% BSA in PBS, pH 7.4) for 1 h at RT. Standard or sample (100 µL) were inserted into each well. In some cases, samples were diluted in dilution reagent (and assigned their respective correction factors in the result analysis) provided by the kit and incubated for 1 h at RT. Then, the plates were washed, and 100 µL of the detection antibody was added for 1 hour. After a new washing step, 100 µL secondary antibody (HRP) conjugate was added for 1 hour at RT. Subsequently, the plates were washed, and 100 µL of substrate (TMB) was added to the wells. The colorimetric reaction was stopped by adding 100 µL of 1 M sulfuric acid and subsequently analyzed using the filter at 450 nm.

### 2.13 Survival

Mice received treatment with Lisinopril for 21 days with flavored gelatin, as described in Section 2.3, and were subsequently infected with 10^5^ PFU of SARS-CoV-2. Treatment continued for more 15 days by gavage. Untreated Infected and Untreated Uninfected received flavored gelatin without the addition of medication and, post-infection, PBS 1x by gavage. Weight loss and clinical manifestations were observed, respecting the humanitarian end, described in Section 2.11, in which case mice were subjected to euthanasia.

### 2.14 Histopathological analysis

On the 6/7 dpi, lungs were harvested and placed in 10% formaldehyde for 24 h and transferred to 70% alcohol, embedded in paraffin, and cut at 5 µm thickness. The deparaffination was made in an oven at 45°C for 40 min followed by xylol for 10 min. Samples were hydrated using alcohol (100%, 80%, and 70%) for 5 min each, then washed in running water and stained with hematoxylin for 3 min. The slides were rewashed, and then the eosin was inserted for 7 min. Subsequently, the dehydration step was carried out using ethyl alcohol (70%, 80%, and 100%), followed by the fixation step. A descriptive table was created with an intensity scale (score from 0 to 4) for histopathological findings.

### 2.15 Statistical analysis

The results were entered into a database in Excel software and analyzed individually using statistical methods appropriate for each type of experiment. Normality was checked using Kolmogorov–Smirnov, D’Agostino–Pearson, and Shapiro–Wilk tests. Student’s T/Multiple *t*-test and ANOVA with Bonferroni multiple comparisons test were used for parametric variables. Mann Whitney and Kruskal Wallis tests were performed for non-parametric data followed by Dunn’s analysis of variance. The Log-rank (Mantel–Cox) test was applied for the survival curves. Differences between groups were considered significant when the *p*-value was <0.05. Graph Prism software version 8.0 was used.

## 3 Results

### 3.1 Lisinopril-treated mice show increased ACE2 levels and higher SARS-CoV-2 viral loads in the lungs

**FIGURE 1:**
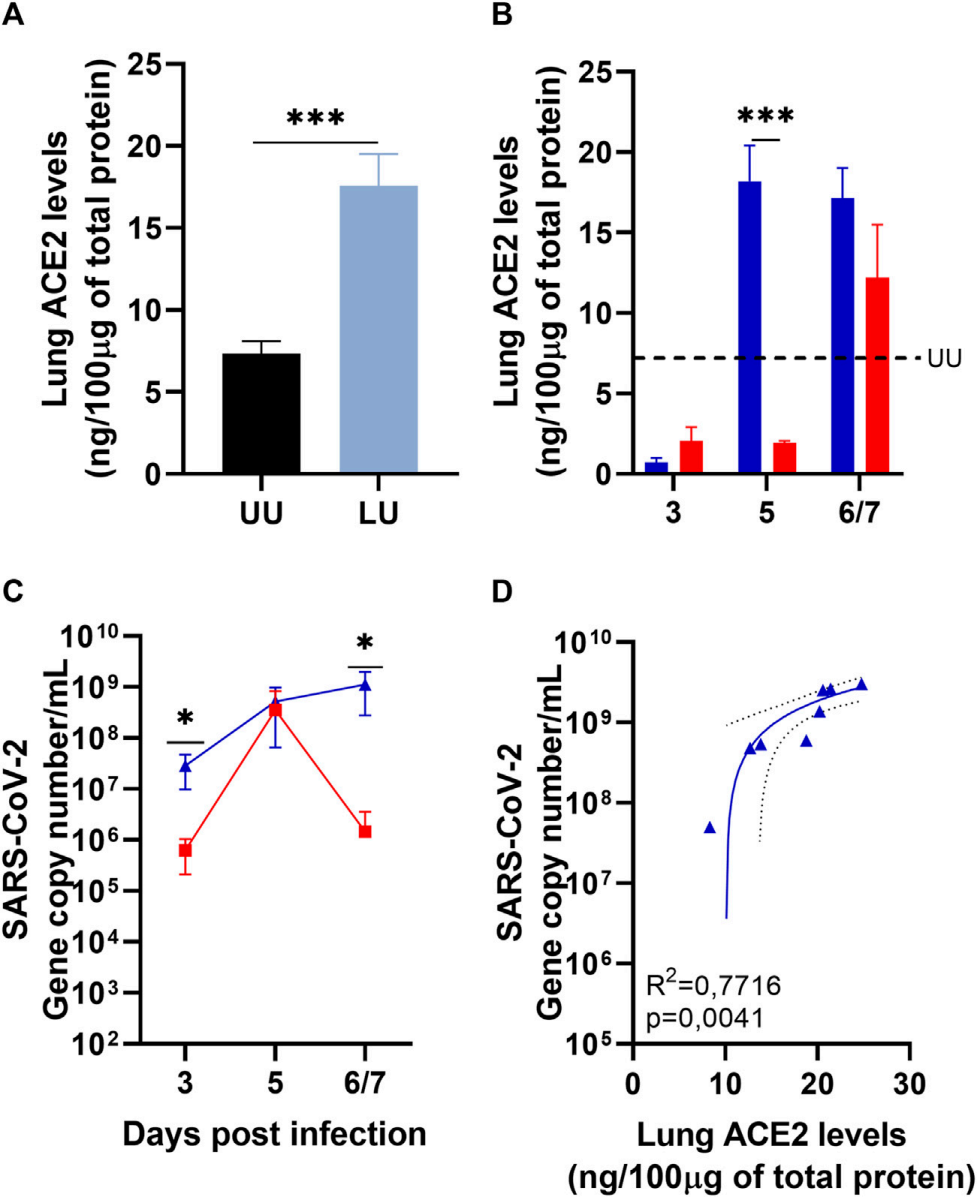
Quantification of lung ACE2 by ELISA and lung viral load by RT-PCR. **(A)** Lung ACE2 in Uninfected mice treated or not with Lisinopril for 21 days: Untreated Uninfected (UU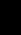) (*n* = 10), Lisinopril-treated Uninfected (LU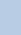) (*n* = 8). **(B)** Lung ACE2 in mice treated or not for 21 days with Lisinopril and then infected with SARS-CoV-2: Lisinopril-treated Infected (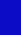) (*n* = 10/timepoint), Untreated Infected (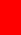) (*n* = 10/timepoint); the mean value for Untreated Uninfected controls (UU) is shown as a dashed line (**-- -**). **(C)** Viral load and **(D)** correlation (linear regression) between viral load and ACE2 levels in Lisinopril-treated Infected mice (6/7 dpi): Lisinopril-treated Infected (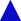) (*n* = 10/timepoint), Untreated Infected (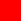) (*n* = 10/timepoint). Data presented as mean ± SEM. Asterisks indicate significance; *p < 0.05, **p < 0.005 and ***p < 0.0005 by ANOVA 2 way with Bonferroni’s multiple comparisons and *t*-test.

The lungs of uninfected mice were analyzed to understand the influence of ACEi treatment on ACE2 levels in humanized transgenic mice treated or not with Lisinopril. As expected, treatment with Lisinopril led to a marked increase in ACE2 levels in the lungs after 21 days of daily drug administration (Figure 1A). The effect was stronger in female than in male mice (Supplementary Figure S1A).

Infection with SARS-CoV-2 led to a sudden reduction in ACE2 levels in the lungs of both Untreated Infected and Lisinopril-treated Infected mice on 3 dpi (Figure 1B). ACE2 levels remained low in the Untreated Infected mice at 5 dpi, but strikingly ACE2 levels recovered to pre-infection levels at 5 dpi in Lisinopril-treated Infected mice. At the last time point (6-7 dpi), ACE2 levels were also recovered in the Untreated Infected mice (Figure 1B), but interestingly female mice showed much higher levels than male mice (Supplementary Figure S1C). No differences were observed between sexes in the Lisinopril-Treated Infected group (Supplementary Figures S1B, S1C).

The higher levels of ACE2 in the lungs of Lisinopril-treated mice seem to have influenced the viral load in these animals. Lisinopril-treated Infected mice showed much higher SARS-CoV-2 copy numbers at 3 and 6/7 dpi than Untreated Infected mice (Figure 1C). No differences were observed between the sexes in the Lisinopril-Treated Infected group and the Untreated Infected group (Supplementary Figures S1D, S1E).

The higher viral load recorded on 3 dpi was not proportional to ACE2 levels; however, at the last time point, we observed a positive correlation between viral load and the quantification of ACE2 in the lung (*R* ^2^ = 0.7716; *p* = 0.0041 (Figure 1D)). There seems to be a correlation between lung ACE2 levels at a given time point and viral load at the subsequent time point. Mice treated with Lisinopril for 21 days showed higher ACE2 levels than untreated mice just before infection. Consequently, the viral load at 3 dpi for Lisinopril-treated Infected mice was much higher than for Untreated Infected mice. At 3 dpi, ACE2 levels plummeted in both groups, treated and untreated, to similar low levels, and viral titers at 5 dpi were also similar in the two groups. At 5 dpi, ACE2 levels remained very low in Untreated Infected mice, leading to a steep decrease in viral load at 6/7 dpi, whereas ACE2 levels soared in Lisinopril-treated Infected mice at 5 dpi, leading to sustained high viral titers at 6/7 dpi, which were 1,000 times higher than those observed in Untreated Infected mice.

### 3.2 Lisinopril does not alter respiratory capacity in experimental COVID-19

**FIGURE 2:**
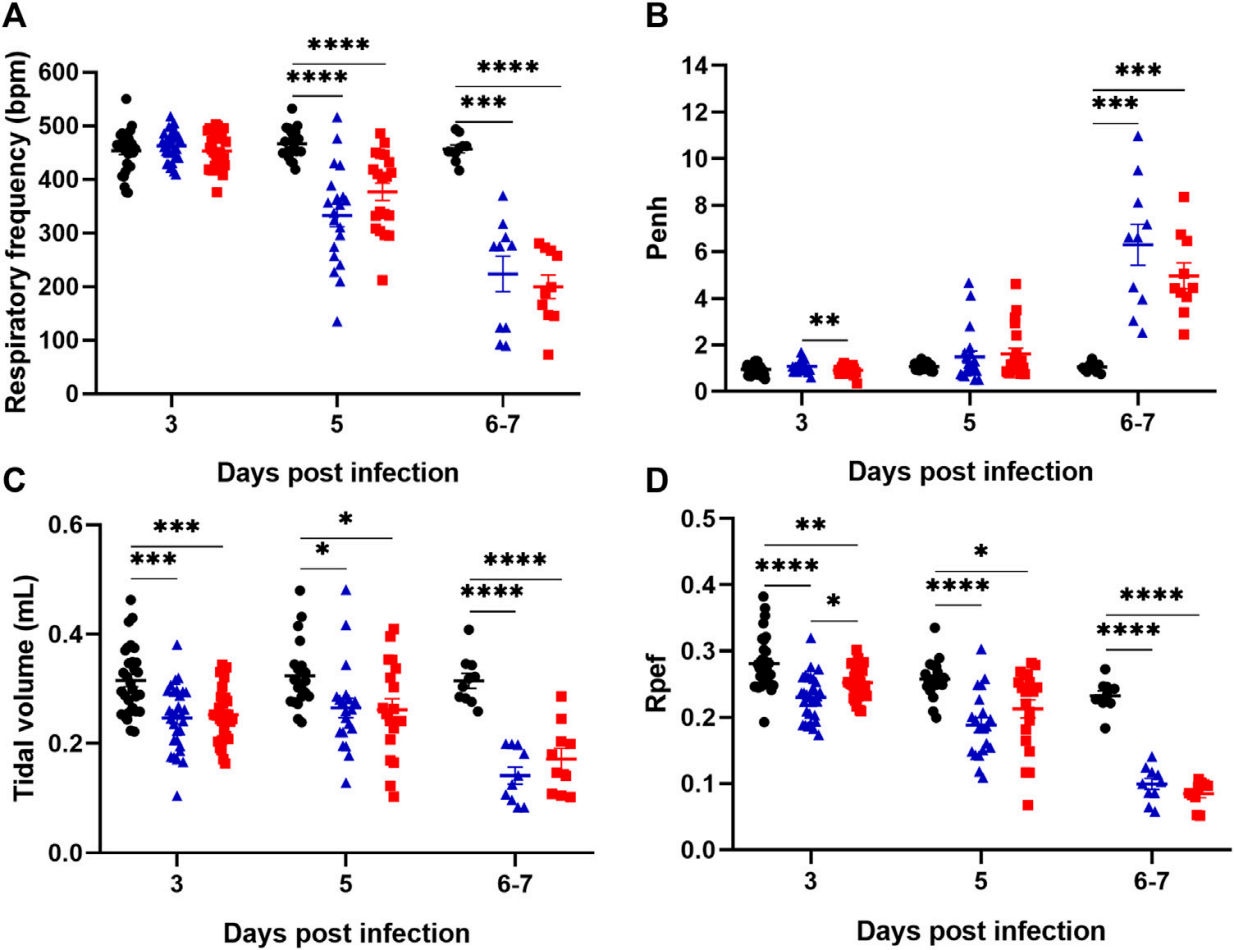
Assessment of lung capacity through the parameters of respiratory frequency **(A)**, Enhanced pause (Penh) **(B)**, tidal volume **(C)** and expiratory flow curve (Rpef) **(D)** in K18 hACE2 mice observed on days 3 (*n* = 30), 5 (n = 20) and 6/7 (*n* = 10) post-infection (dpi). Data presented as mean ± SEM. Asterisks indicate significance: *p < 0.05, **p < 0.005, ***p < 0.0005 and ****p < 0.0001 by 2-way ANOVA with multiple comparisons by Bonferroni. Representative results from three independent experiments. Untreated Uninfected (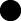), Lisinopril-treated Infected (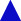), Untreated Infected (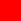).

Lung capacity was measured on days 3, 5, and 6/7 post-infection. Irrespective of whether mice were treated or not with Lisinopril, infection with SARS-CoV-2 led to worsened respiratory capacity. There was a reduction in Respiratory frequency (RF) at 3, 5, and 6/7 dpi, and in tidal volume and expiratory flow curve (Rpef) at 5 and 6/7 dpi between the Untreated Infected and Lisinopril-treated Infected groups compared to the Untreated Uninfected (Figures 2A,C,D). Concerning enhanced pause (Penh), an increase was observed at 6/7 dpi in both infected groups compared to Untreated Uninfected (Figure 2B). All respiratory capacity indicators were much worse at 6/7 dpi than in the previous time points.

These data indicate that SARS-CoV-2-infected mice show increasingly deteriorated lung capacity during infection. However, Lisinopril treatment does not seem to impact on the respiratory capacity outcome. Respiratory parameters were also analyzed separately for males and females. In general, the results indicated no significant differences in the respiratory pattern between sexes, except for a few measurements at specific time points in the Untreated Infected group (e.g., respiratory frequency at 3 dpi and tidal volume at 3 and 6/7 dpi) (Supplementary Figure S2).

### 3.3 Treatment with lisinopril has a transient impact on the clinical manifestations of experimental COVID-19

**FIGURE 3:**
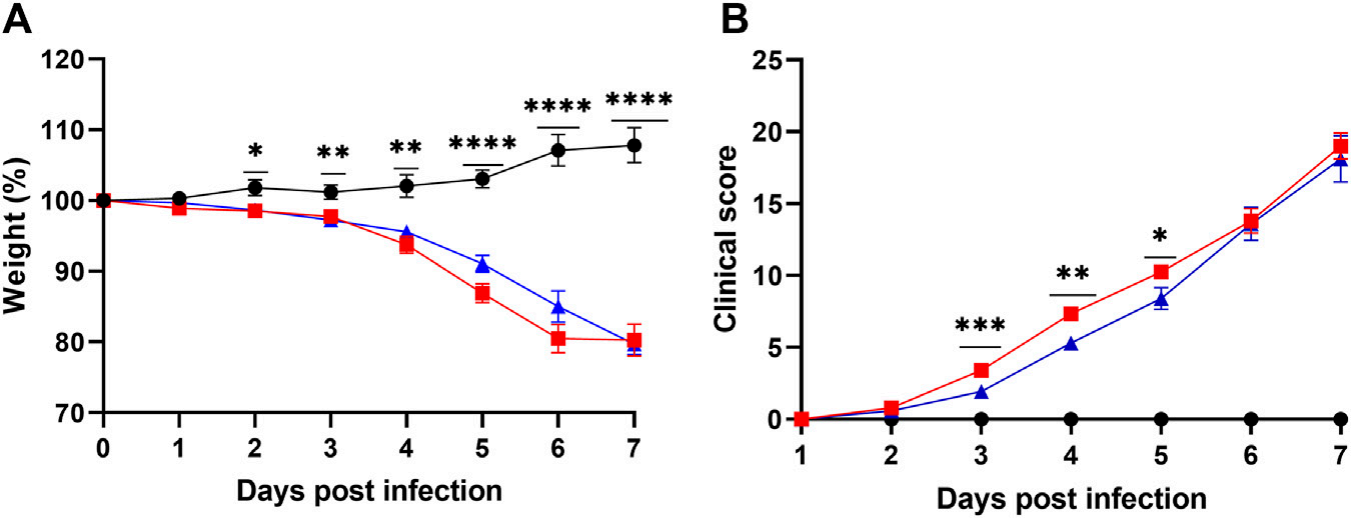
**(A)** Body weight loss (%) and **(B)** clinical score (daily sum) of K18 hACE2 Untreated (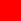; *n* = 30) and Lisinopril-treated (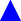; *n* = 30) mice during SARS-CoV-2 infection, compared to the Untreated Uninfected (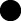; *n* = 30) mice. The Untreated Uninfected group did not present clinical signs or weight loss. Representative results from three independent experiments. Asterisks indicate significance: *p < 0.05, **p < 0.005, ***p < 0.0005 and ****p < 0.0001 by 2-way ANOVA followed by multiple comparisons by Bonferroni.

Untreated Infected mice showed progressive weight loss reaching out 20% by day 6 of infection, and Lisinopril-treated Infected mice showed a similar outcome (Figure 3A). Significant weight loss in both infected groups in relation to Untreated Uninfected was observed from day 2 of infection. Untreated Infected mice also showed worsening of the clinical score, which deteriorated from day 3 (Figure 3B). Overall, weight loss and deteriorating clinical scores were similar between Untreated Infected and Lisinopril-treated Infected mice. *Post-hoc* analysis showed that Lisinopril-treated Infected mice presented slightly improved weight at 5 dpi and better clinical scores at 3, 4, and 5 dpi; however, in the end results (6 and 7 dpi), the clinical scores were not different between the two groups. No differences in weight loss and clinical score were observed between male and female mice (Supplementary Figure S3).

### 3.4 Lisinopril treatment decreases the levels of pro-inflammatory cytokines in experimental COVID-19

**FIGURE 4:**
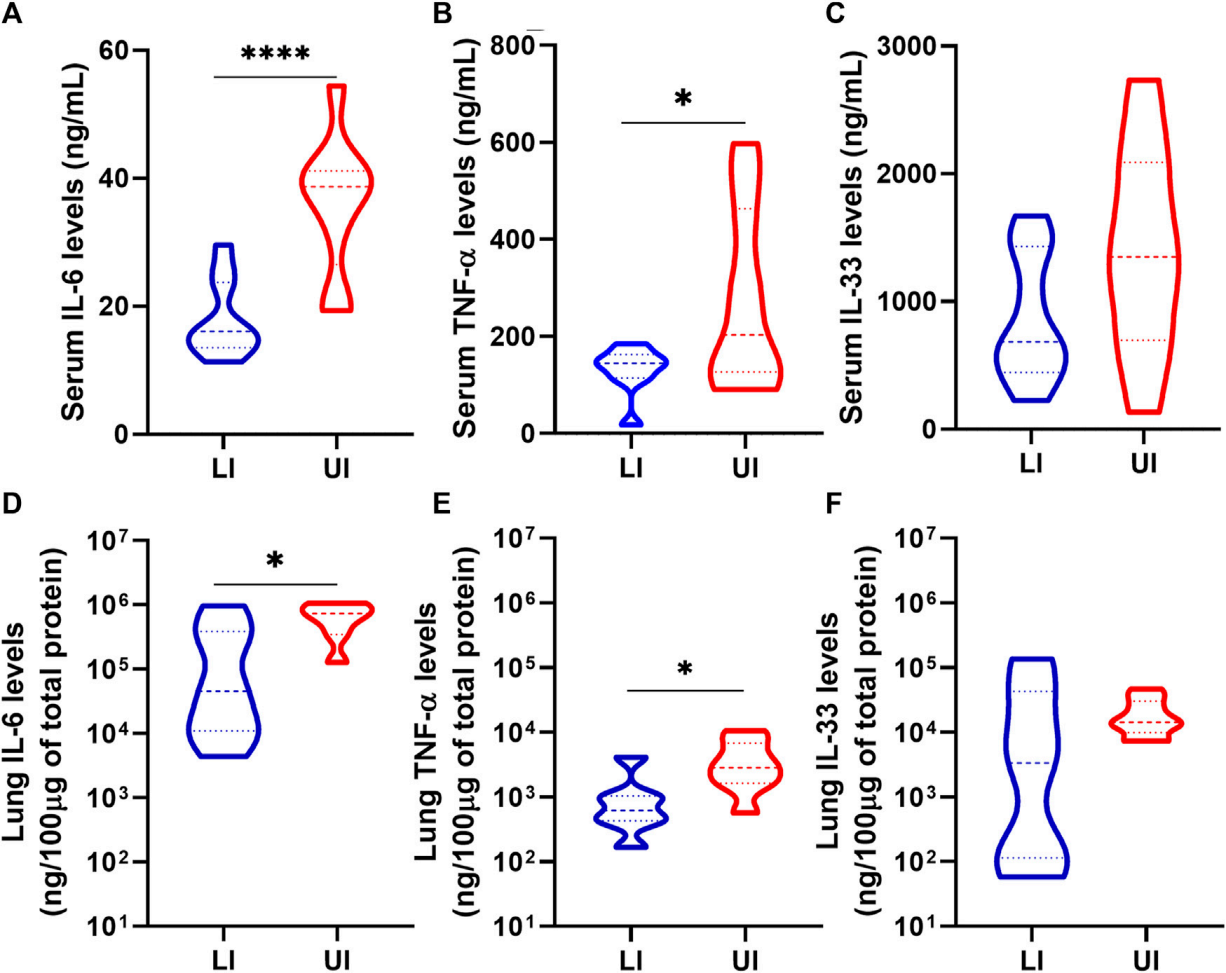
Analysis of IL-6 **(A** and **D)**, TNF-α **(B and E)** and IL-33 **(C and F)** in serum and lung of Lisinopril-treated Infected (LI; *n* = 10) and Untreated Infected (UI, *n* = 10) mice on 6/7 dpi. Untreated Uninfected controls showed values below 0.5 ng/mL for the three cytokines. Data presented as mean ± SEM. Asterisks **indicate** significance: *p < 0.05 and ***p < 0.0005 by *t*-test or Mann-Whitney test.

The levels of interleukin-6 (IL-6), tumor necrosis factor alpha (TNF-α) and IL-33 were measured in the serum and lungs at 6/7 dpi. As expected, Untreated Infected mice showed high levels of the three pro-inflammatory cytokines in the serum and the lungs (Figure 4). Treatment with Lisinopril led to a marked reduction in the levels of IL-6 and TNF-α both in the serum and lungs (Figures 4A,B,D,E). Despite overall lower levels, a significant reduction in IL-33 was not observed (Figures 4C,F). No differences were observed in serum and lung cytokine levels between the sexes (Supplementary Figure S4).

### 3.5 Lisinopril does not affect lung histopathological changes and outcome of SARS-CoV-2 infection

**FIGURE 5:**
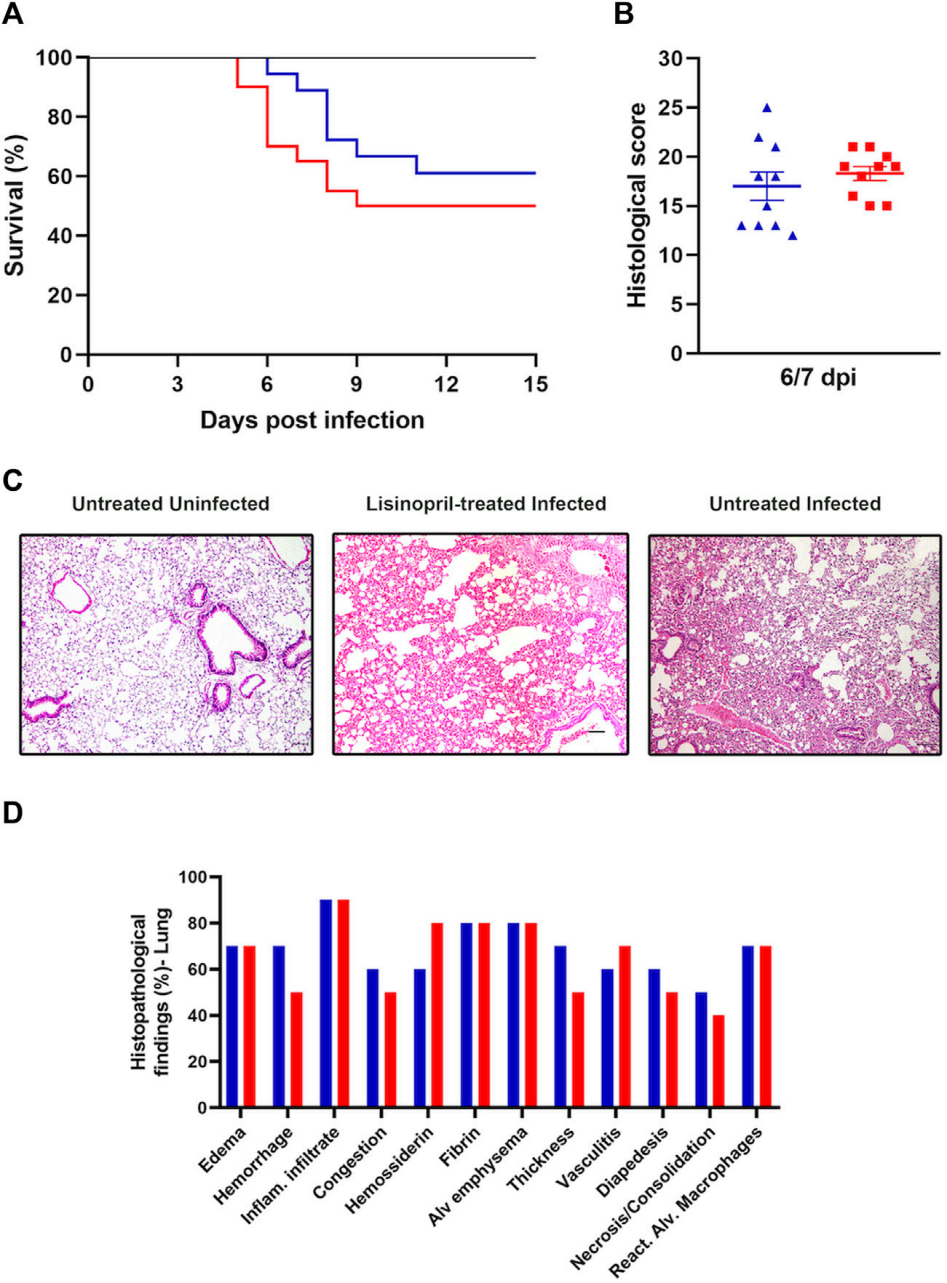
**(A)** Survival curve of Untreated (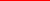; *n* = 18) and Lisinopril-treated (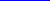; *n* = 20) K18-hACE2 mice after infection with SARS-CoV-2; the Untreated Uninfected (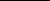; *n* = 6) did not show mortality. The survival Log-rank (Mantel–Cox) test was applied. **(B–D)** Histopathological analysis of lungs on 6/7 dpi. **(B)** histological scores of Lisinopril-treated Infected (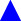; *n* = 10) and Untreated Infected (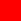; *n* = 18) K18-hACE2 mice. Results obtained from the sum of the degrees of intensity. Data presented as mean ± SEM. Analysis performed by *t*-Test. **(C)** Representative histopathological images from lungs. Magnification = ×100. **(D)** Frequency of histopathological findings at 6/7 dpi in Lisinopril-treated Infected (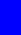; *n* = 10) and Untreated Infected (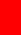; *n* = 10) mice.

Survival and lung histopathological changes were verified to analyze the effect of treatment with Lisinopril on the outcome of SARS-CoV-2 infection. Infection with SARS-CoV-2 in untreated mice led to a 50% mortality rate, with deaths occurring between days 5–9, whereas treatment with Lisinopril resulted in a lower (39%) and delayed (days 6–11) mortality (Figure 5A), but these outcomes were not statistically different. The outcomes among female and male mice are shown in Supplementary Figures S5A, S5B.

Lung histopathology was analyzed at 6/7 dpi and classified according to the sum of the degrees of intensity and frequency (%) of histopathological changes. As expected, lung injuries in SARS-CoV-2-infected mice were severe and determined the cause of death. Lisinopril treatment had no apparent impact on this outcome. According to the analysis of the intensity of histopathological findings in lung tissue (0–4), no differences were observed in the Untreated Infected and Lisinopril-treated Infected groups (Figure 5B). Inflammatory infiltrate was the most frequent finding in both the Untreated Infected and Lisinopril-treated Infected groups (90%), followed by alveolar emphysema and the presence of fibrin (80%). Edema and reactive alveolar macrophages were present in 70% of the tissues analyzed in both groups. Furthermore, hemorrhage and thickening of the alveolar septa were observed in 70% of the lungs, and congestion in 60% was more frequent in the Lisinopril-treated Infected group (50%). Hemosiderin was more frequent in Untreated Infected mice (80%). These results are represented in Figures 5C,D.

## 4 Discussion

The discussion about the effects of treatment on patients revolves around the possible protective or deleterious effects on RAS imbalance. Due to the urgency of information during a pandemic, many clinical trials and prospective studies were conducted to fill information gaps. However, more exploratory studies under controlled conditions allowed by animal experimental models of COVID-19 under ACEi interference can bring precious information regarding mechanisms of physiopathogenesis involved in the process (Liu et al., 2020; Patel and Verma, 2020; Baral et al., 2021). The present study used a transgenic humanized animal model widely applied for studies of the pathogenesis of COVID-19, simulating continuous treatment with Lisinopril for 21 days before infection with 10^5^ PFU of SARS-CoV-2, maintaining treatment for up to 7 days post-infection.

The key, novel findings of this study are the demonstration that Lisinopril treatment leads to increased expression of ACE2 in the lungs, the main target organ of SARS-CoV-2, and this effect seems to be a key element making these animals susceptible to higher viral loads at the earlier and later stages of the infection. On the other hand, Lisinopril treatment largely prevented the occurrence of high levels of the pro-inflammatory cytokines IL-6 and TNF-α. These two opposite effects, deleterious in terms of viral load and beneficial in terms of limited inflammation, may help to explain the lack of major differences of outcome, whether considering clinical evolution, respiratory capacity, lung damage or survival, between Lisinopril-treated and Untreated animals (Zhang et al., 2020; Meng et al., 2020).

Quantification of ACE2 in healthy mice (Untreated Uninfected) compared to animals that received Lisinopril (Lisinopril-treated Uninfected) was essential to understanding the scenario under virus interference. Our results demonstrated that ACE2 in the lungs increase after 21 days of treatment, similar to the findings described by Brooks and colleagues in C57BL/6 mice (Brooks et al., 2022). Interestingly, this effect was stronger in female than in male mice, but did not seem to influence post-infection outcomes (Lisinopril-treated female mice showed patterns of lung ACE2 levels and viral load similar to male mice after infection). The lung is the most important organ for the development of COVID-19. A high viral load in this tissue was expected, as observed in the Untreated Infected group, with viral replication peaking at the 5 dpi. However, the Lisinopril-treated Infected mice showed a progressive increase in viral load over time correlated with the quantification of ACE2, confirming the initial hypothesis about the duality of events caused by the interference of an ACEi in the RAS of these animals. In this case, Lisinopril upregulated ACE2, increasing viral replication.

The high viral load recorded at 3 dpi was not correlated with the quantification of ACE2; however, at the last time point, we observed that the variables are strongly correlated (*R* ^2^ = 0.7716; *p* = 0.0041). This result suggests that the Lisinopril-treated Infected group increases in viral load due to the continuous use of Lisinopril by favoring the alternative RAS pathway.

Replication of the virus is the triggering event in COVID-19 pathogenesis, and therefore an increased and persistent viral load points to a more severe outcome. However, the results of weight loss, clinical score, and lung capacity do not indicate that this increase in viral load interfered with the outcome of infection compared to the untreated infected group. The nature and potency of the host response, particularly in terms of inflammation and coagulation, have been well-established as critical elements of COVID-19 severity (Brandão et al., 2020; Velavan et al., 2021; Veiga and Cavalcanti, 2023). Indeed, in COVID-19, high levels of IL-6 and TNF-α were associated with a greater chance of developing respiratory failure and contributed to the formation of fibrinogen and other factors in the coagulation cascade (Lipworth et al., 2020; Vultaggio et al., 2020; Lazzaroni et al., 2021), and IL-33 has been shown to be upregulated in COVID-19 patients and strongly associated with poor outcomes (Gao et al., 2022). Our results showed that SARS-CoV-2-infected hACE2 mice had increased serum and lung levels of the three cytokines, and Lisinopril treatment reduced serum IL-6 and TNF-α but not IL-33 levels. A limited inflammatory response has been shown to be important for improved prognosis and for preventing the development of long/severe COVID-19, as demonstrated in patients (Li et al., 2020; Liu et al., 2020; 2023; Meng et al., 2020; Naicker et al., 2020; Yonas et al., 2020; Giannitrapani et al., 2023; Yin et al., 2023). In addition, IL-6 analysis indicated a decrease in inflammatory action, resulting from the blockade of the AT1R receptor by the drug’s action, favoring the Ang (1–9) axis to the Mas receptor (Vaduganathan et al., 2020; Shekhawat et al., 2021). Also, ACEi alters the production of cytokines and has effects on the recruitment of monocytes and macrophages to the site of inflammation (Felkle et al., 2022), with protective effects on heart and kidney diseases (Krysiak and Okopień, 2012; Abdel-Wahab et al., 2014; Haas et al., 2019; Tesch et al., 2019; Tan et al., 2024). Therefore, despite boosting viral replication, the anti-inflammatory action of the Lisinopril treatment probably acted as a compensatory mechanism, resulting in no major differences in terms of outcome compared to the untreated animals.

As any animal model of human disease, the K18-hACE2 mice has a number of limitations. Being under the control of cytokeratin K18 promoter, hACE2 expression is limited to epithelial cells and, therefore, does not reflect the complexity of the hACE2 expression—and hence of SARS-CoV-2 propagation—in a number of different tissues in humans. But, since in these mice the hACE2 is expressed in sites that are critical for both virus replication and pathology in human COVID-19, such as upper respiratory airways, lungs, kidneys and intestines, it replicates consistently—though not perfectly—the human disease. It also needs to be emphasized that ACE2 plays major roles in COVID-19 not only as the receptor for the virus but also because its downregulation during infection probably has marked impacts on cardiovascular and coagulation functions, aggravating the disease. In this regard, ACE inhibitors upregulate not only the hACE2 in the transgenic mice, but also the native mouse ACE2 (Brooks et al., 2022).

As such, increased ACE2 expression induced by lisinopril may help to explain a compensatory effect against increased viral load. As mentioned, the sharp decrease in ACE2 expression in the lungs and other tissues by SARS-CoV-2 infection is highly detrimental to vascular health, generating a pro-constrictory and pro-coagulation environment, which plays a central role in COVID-19 pathogenesis (Ye et al., 2020; Li et al., 2021). Lisinopril-treated animals showed not only higher ACE2 levels before infection but also showed a fast recovery (5 dpi) of ACE2 levels after initial (3 dpi) ACE2 exhaustion. This normalization of ACE2 expression earlier in the infection may have contributed to prevent or diminish systemic deleterious events such as widespread coagulation as well as local tissue damage in face of higher viral load.

Indeed, the Lisinopril-treated Infected group showed no worse performance in terms of clinical condition, respiratory capacity, lung damage and mortality compared to the Untreated Infected group. A retrospective study (Yang et al., 2020) found a lower proportion of critically ill patients and a lower mortality rate, while systematic reviews/meta-analyses did not provide evidence of a significant association between ACEi/ARB treatment and COVID-19 mortality. Additionally, another study (Huang et al., 2021) showed a reduction in the inflammatory profile in patients treated with ACEI/ARB and Zhang et al. found no association with a higher risk of severe infection (Zhang et al., 2020).

A few differences between male and female mice were observed in this study. Infection by SARS-CoV-2 in untreated mice led to slightly worse performance in female compared to male mice for some respiratory function parameters (e.g., respiratory frequency, Penh and tidal volume at 3 dpi and tidal volume at 6/7 dpi). Untreated infected female mice had also much higher levels of lung ACE2 at 6/7 dpi. In the treated group, Lisinopril led to higher levels of lung ACE2 in female than in male mice, but this effect did not result in marked differences in outcomes between male and female mice. Overall, lung ACE2 levels were markedly affected by sex, but otherwise sex differences seemed to be small and studies with bigger sample sizes and additional endpoints may be necessary to unveil significant sex-related factors influencing SARS-CoV-2 infection outcomes in these scenarios.

It is important to emphasize that, in this study, SARS-CoV-2 infection was carried out in health, young adult mice. ACEi such as Lisinopril are used by patients with chronic vascular diseases such as hypertension, which are associated with severe outcomes in COVID-19 (Khashkhusha et al., 2020; Rashedi et al., 2020). Fear that ACEi might increase the probability of severe outcomes of SARS-CoV-2 infection due to increased ACE2 expression led to considerations of interrupting ACEi use in COVID-19. However, clinical data suggest that continuing treatment is the best alternative (de Abajo et al., 2021; Macedo et al., 2022). In addition, our findings show that continued treatment during SARS-CoV-2 infection may be protective against the development of severe forms of COVID-19. Studies using similar approaches in models of hypertension with hACE2 mice should provide more definitive indication that, in a chronic disease scenario, the benefits of ACEi treatment during COVID-19 may be far more evident.

In summary, the present study brings evidence that ACEi (Lisinopril) shows both deleterious (higher viral loads) and beneficial (anti-inflammatory and probably anti-constrictory and anti-coagulant) effects in experimental COVID-19. These effects seem to compensate each other, resulting in marginal beneficial effects in terms of outcome for Lisinopril-treated animals.

## Data Availability

The raw data supporting the conclusion of this article will be made available by the authors, without undue reservation.

## Supplementary material

SUPPLEMENTARY FIGURE S1 Quantification of ACE2 levels by sex in lungs of uninfected mice treated with Lisinopril for 21 days compared to Untreated Uninfected group. *n* = 4 males and 4 females per group **(A)**. Quantification of ACE2 levels **(B and C)** and viral load **(D and E)** by sex in Lisinopril-treated (*n* = 5 males and 5 females) and Untreated Infected (*n* = 5 males and 5 females) mice on 6/7 dpi. Data presented as mean ± SEM. Asterisks indicate significance; **p* < 0.05, ****p* < 0.0005 and *****p* < 0.0001, by ANOVA two way with Bonferroni’s multiple comparisons, respectively.

SUPPLEMENTARY FIGURE S2 Assessment of lung capacity through the parameters of Respiratory frequency **(A, B and C)**, Enhanced pause (Penh) **(D, E and F)**, Tidal volume **(G, H and I)** and expiratory flow curve (Rpef) **(J, K and L)** in male and female K18 hACE2 mice observed on days 3, 5, and 6/7 post-infection. Data presented as mean ± SEM. Asterisks indicate significance; **p* < 0.05, ***p* < 0.005, and ****p* < 0.0005 by 2-way ANOVA with multiple Bonferroni comparisons. 3 dpi *n* = 15, 5 dpi *n* = 10 and 6/7 *n* = 5 animals per group. Representative results from three independent experiments. Untreated Uninfected ( ● ), Lisinopril-treated Infected ( ▲ ), Untreated Infected ( ■ ).

SUPPLEMENTARY FIGURE S3 The graphics represent weight loss and clinical score (daily sum) between males and females of K18 hACE2 mice infected with SARS-CoV-2 by group Lisinopril-treated infected **(A and C)** and Untreated Infected **(B and D)**. Data presented as mean ± standard error of the mean (SEM), by Multiples T test.

SUPPLEMENTARY FIGURE S4 Analysis of IL-6, TNF-α and IL-33 in serum and lung of Lisinopril-treated Infected group **(A and C)** and Untreated Infected group **(B and D)** on 6/7 dpi. *n* = 5 mice per group. Performed by T test or Mann Whitney test.

SUPPLEMENTARY FIGURE S5 Survival curve of Lisinopril-treated Infected **(A)** and Untreated Infected groups **(B)**. The survival Log-rank (Mantel–Cox) test was applied and no differences were observed between the sexes. Lisinopril-treated Infected *n* = 18 (9 males and 9 females) and Untreated Infected *n* = 20 (10 males and 10 females).
