# Supplementary figures and images for: Lisinopril increases lung ACE2 levels and SARS-CoV-2 viral load and decreases inflammation but not disease severity in experimental COVID-19
Source: Front Pharmacol. 2024 Jul 12;15:1414406. doi: 10.3389/fphar.2024.1414406 (PMC11282493; doi:10.3389/fphar.2024.1414406)

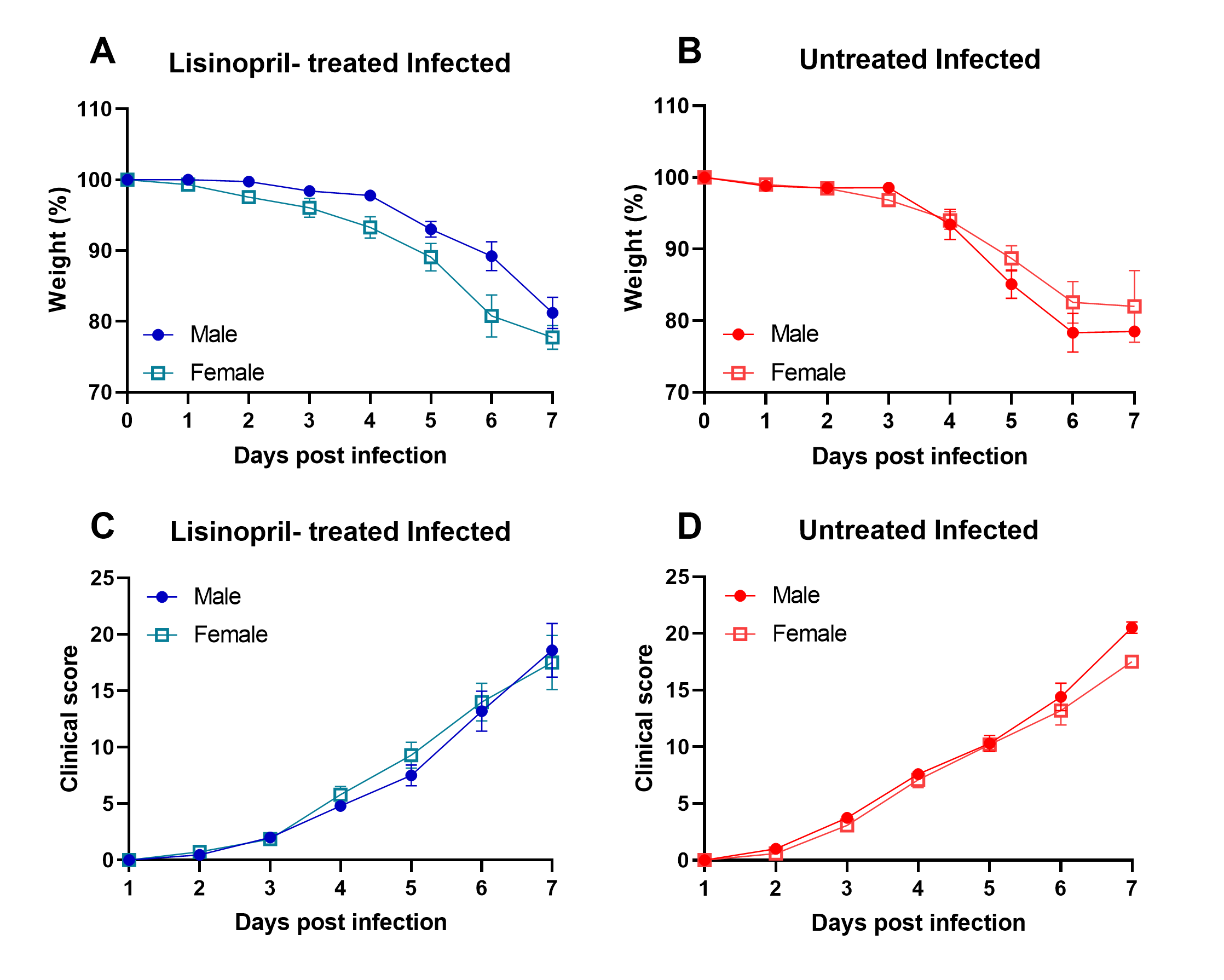

Supplement: Supplementary file 1 [file Image3.TIF]

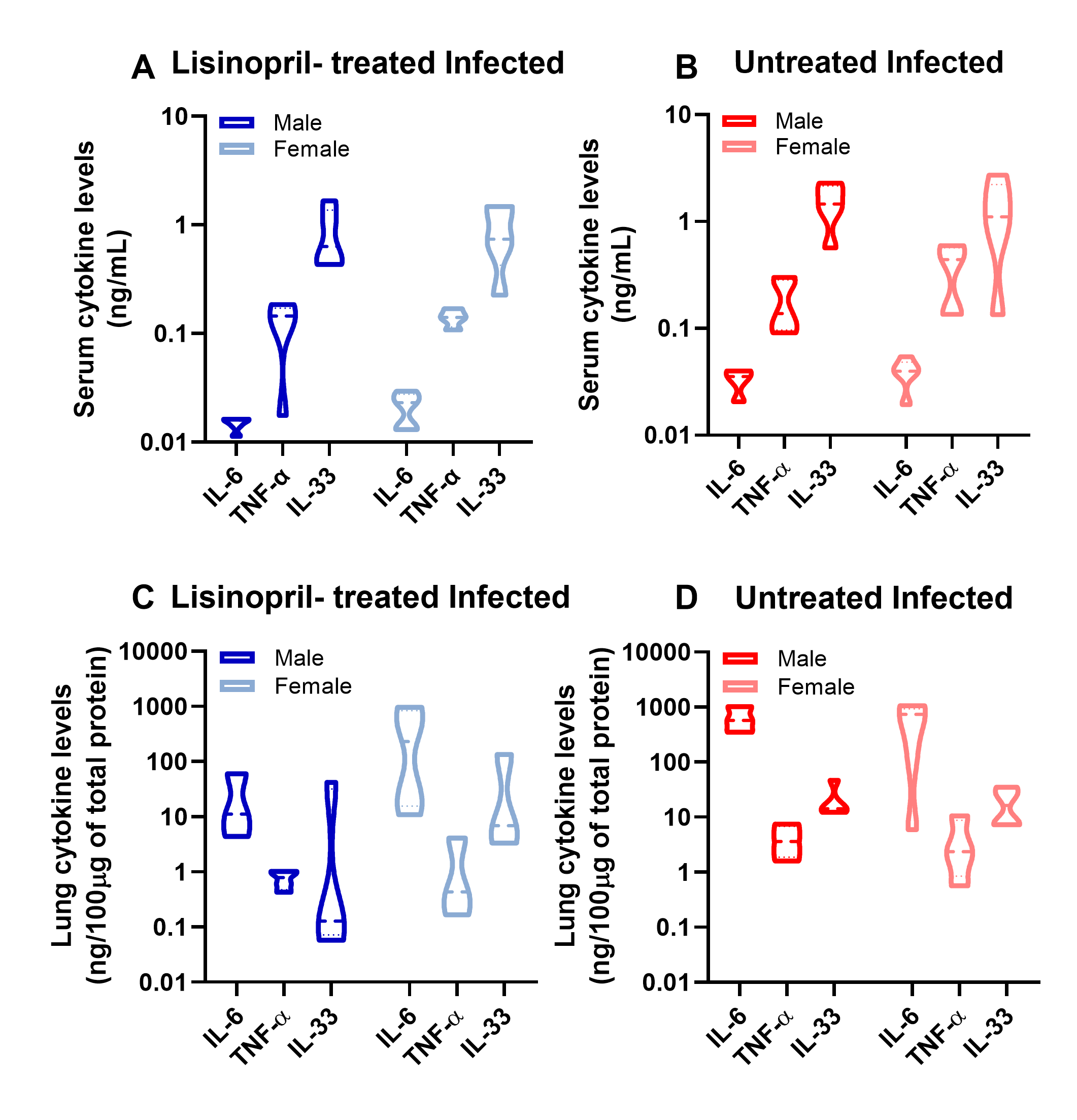

Supplement: Supplementary file 2 [file Image4.TIF]

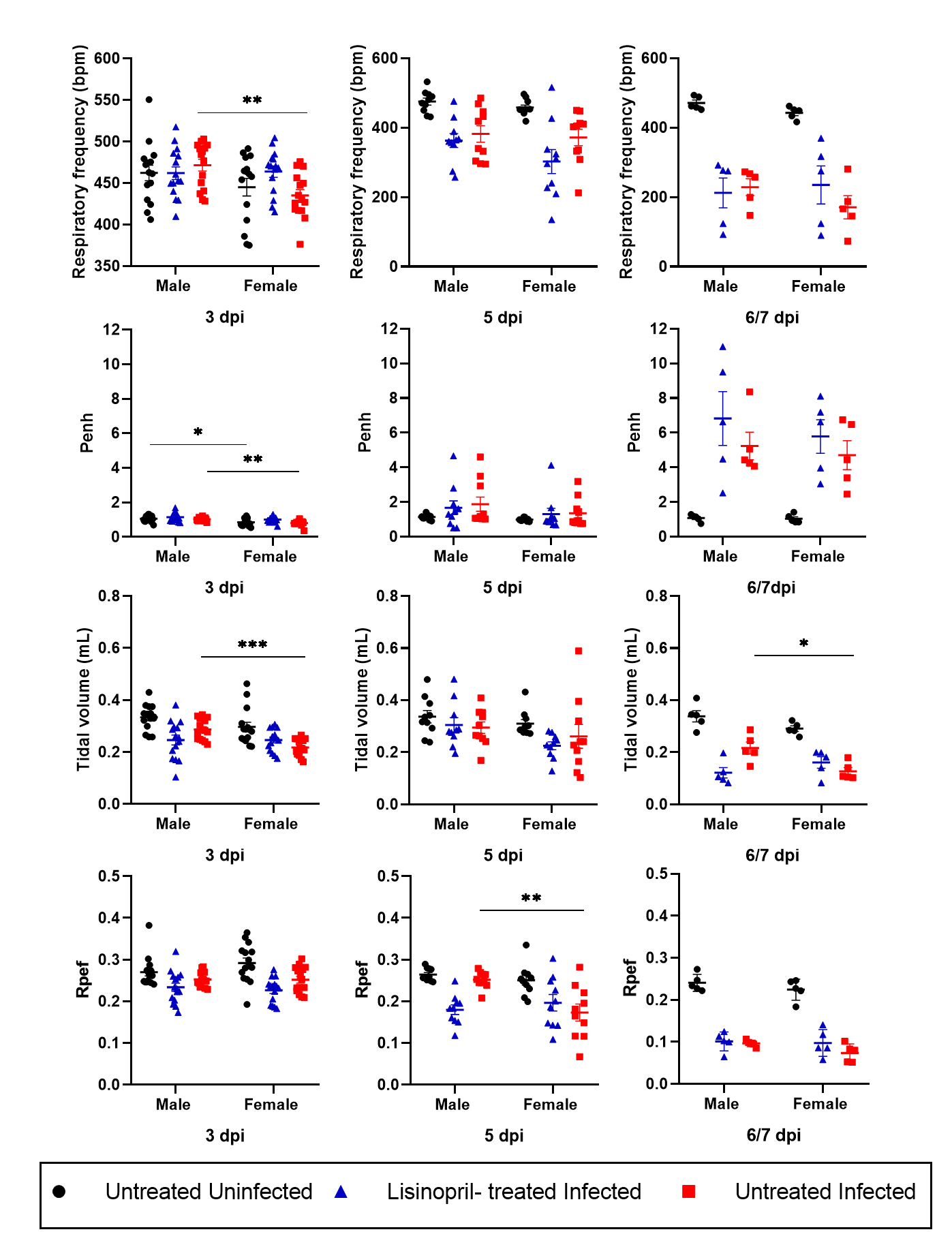

Supplement: Supplementary file 3 [file Image2.TIF]

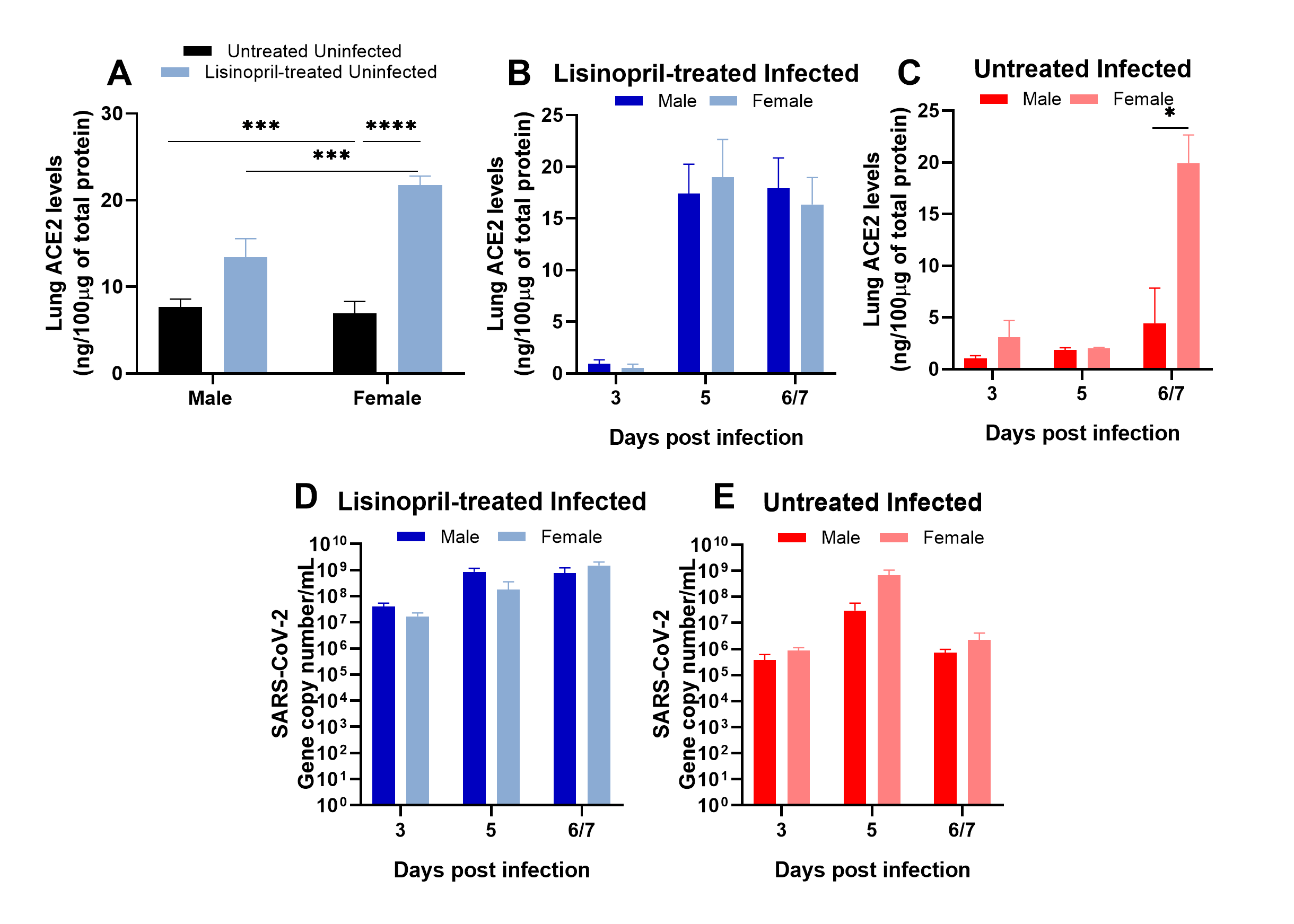

Supplement: Supplementary file 4 [file Image1.TIF]

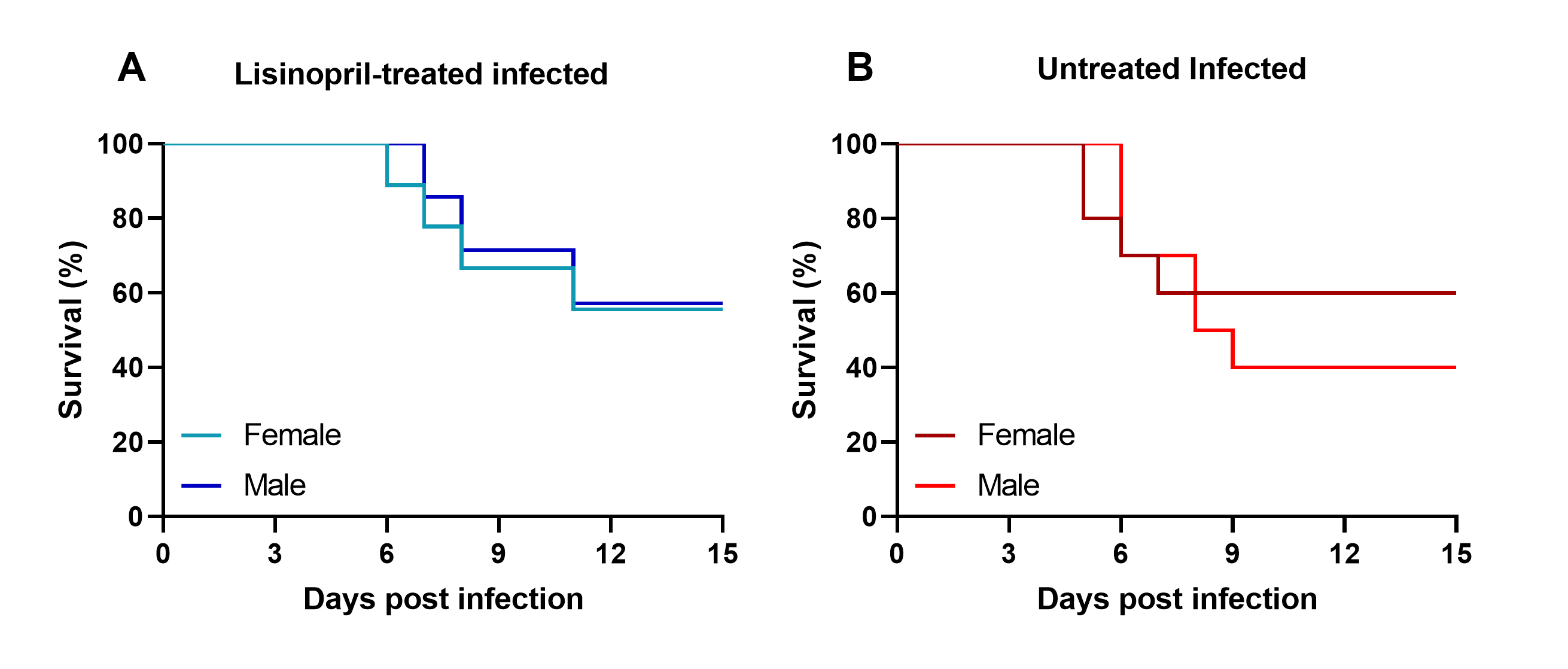

Supplement: Supplementary file 5 [file Image5.TIF]
